# Role of mechanosensitive channel Piezo1 protein in intestinal inflammation regulation: A potential target

**DOI:** 10.1096/fj.202401323R

**Published:** 2024-10-19

**Authors:** Qinlei Jiang, Zhenyu Li, Dan Dang, Jiaqi Wei, Hui Wu

**Affiliations:** ^1^ Department of Neonatology, Children's Medical Center The First Hospital of Jilin University Changchun Jilin People's Republic of China

**Keywords:** immune system, intestinal barrier, intestinal inflammation, Piezo1

## Abstract

The intestine is a hollow tract that primarily transports and digests food. It often encounters mechanical forces and exotic threats, resulting in increased intestinal inflammation attributed to the consistent threat of foreign pathogens. Piezo1, a mechanosensitive ion channel, is distributed broadly and abundantly in the intestinal tissue. It transduces mechanical signals into electrochemical signals and participates in many critical life activities, such as proliferation, differentiation, cell apoptosis, immune cell activation, and migration. Its effect on inflammation has been discussed in detail in systems, such as musculoskeletal (osteoarthritis) and cardiac (myocarditis), but the effects on intestinal inflammation remain unelucidated. Piezo1 regulates mucosal layer and epithelial barrier homeostasis during the complex intestinal handling of foreign antigens and tissue trauma. It initiates and spreads immune responses and causes distant effects of inflammation in the vascular and lymphatic systems, but reports of the effects of Piezo1 in intestinal inflammation are scarce. Therefore, this study aimed to discuss the role of Piezo1 in intestinal inflammation and explore novel therapeutic targets.

## Graphical abstract

Piezo1, a mechanosensitive ion channel, is distributed broadly and abundantly in the intestinal tissue. In the intestines, Piezo1 regulates mucosal layer and epithelial barrier homeostasis during the complex intestinal handling of foreign antigens and tissue trauma. It initiates and spreads immune responses and causes distant effects of inflammation in the vascular and lymphatic systems. This review concludes recent research and discusses the potential role of Piezo1 in intestinal inflammation.

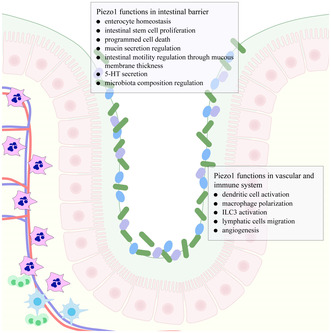

**Abbreviations**
5‐HT: 5‐hydroxytryptamine
CaMK: calmodulin‐dependent kinase
CD: cluster of differentiation
DAMP: damage‐associated molecular pattern
DSS: dextran sulfate sodium
EC cells: enterochromaffin cells
ECM: extracellular matrix
EMT: epithelial‐mesenchymal transition
ERK: extracellular signal‐regulated protein kinases
F/B: *Firmicutes/Bacteroidota*
FAM: fecal‐associated microbiota
H3K9me3: histone 3 lysine 9‐trimethylation
HIF: hypoxia‐inducible factor
IBD: inflammatory bowel diseases
IL: interleukin
ILC3: group 3 innate lymphoid cell
LPS: lipopolysaccharide
MAM: mucosa‐associated microbiota
MUC: Mucin
PAMP: pathogen‐associated molecular pattern
ROS: reactive oxygen species
ssRNA: single‐stranded ribonucleic acid
TCR: T‐cell receptor
TJ: tight junction
TLR: toll‐like receptors

## 1 INTRODUCTION

The intestinal tract is the largest hollow organ of the body; it undergoes constant motor activities, including reflex, segmentation, and migratory movements to continuously transport, digest, and absorb food.^1^, ^2^ Consequently, various mechanical forces—including tension, compression, shear stress, and pressure—act on the intestinal tract. These forces are essential in maintaining normal functions and adapting to complex foreign substances in the lumen. Notably, nearly all intestinal cell types respond to mechanical forces, including intracellular forces like membrane distortion and deformation, volume changes, and extracellular forces such as stretch, tension, and shear stress.^1^, ^3^, ^4^, ^5^, ^6^ Investigating mechanosensitive ion channels that transduce mechanical signals into electrochemical signals is crucial for understanding how mechanical forces affect cellular activities.

Piezo proteins have attracted significant attention since their identification by Coste et al. in 2010^7^; they are mechanosensitive, non‐selective cationic channels, particularly affined to Ca^2+^,^8^ an essential second messenger. They are highly expressed throughout the entire digestive tract and are present in many different intestinal cell types.^9^ Notably, Piezo1 is expressed widely, even in nonsensory tissues and cells including endothelial cells, epithelial cells, and adipocytes, whereas Piezo2 is typically expressed in tissues responding to physical touch.^10^ Piezo1 comprises three parts: peripheral N‐terminal propeller blades that sense mechanical stimulation, beam, and anchor domain for conducting mechanical signals, and the C‐terminal central pore, which facilitates ion transport^11^ and has a specific affinity to Ca^2+^.^7^, ^12^ Given these characteristics, Piezo1 may regulate intracellular and extracellular Ca^2+^ concentrations, alter membrane potential to transmit information between cellular internal and external environments or between cells, and mediate Ca^2+^ influx, which activates various signaling pathways, including p53 and extracellular signal‐regulated protein kinases 1 and 2 (ERK1/2) pathways.^13^

The intestine has a complex environment that continuously encounters foreign antigens. Thus, it is crucial to facilitate precise, suitable, and controllable inflammatory responses to protect against pathogens without inducing autoimmune or exaggerated immune responses simultaneously. Since their discovery, Piezo proteins have been implicated in various biological processes related to inflammation (Table 1), including epithelial homeostasis, stem cell proliferation, macrophage activation and polarization, and the regulation of programmed cell death.^9^, ^26^, ^27^, ^28^ This review focuses on the role and regulation of Piezo1 in various causes and manifestations of intestinal inflammation and explores future perspectives.

**TABLE 1:** Piezo proteins' cellular effects in intestinal cell.

Cell types	Cellular effects	Main finding	Species	Interventions	Reference
Epithelial cells	Extrusion	Knockdown of Piezo1 causes accumulation of excessive epithelial cells, implying Piezo1 regulates live cell extrusion through sphingosine 1‐phosphate (S1P) signaling pathway and ROCK‐dependent myosin contraction.	Zebra fish	Overcrowding strain, photo‐cleavable morpholino to knockdown Piezo1	[14]
	Proliferation	Piezo1 activated by stretch causes calcium influx that activates ERK1/2‐MEK1/2 pathway and transits the G2 to the mitosis phase.	Zebra fish, dog	Stretch, Piezo1 siRNA or photo‐cleavable morpholino	[15]
	Apoptosis	Piezo1 activation causes Ca^2+^ overload and increases cytotoxicity.	Mouse	Piezo1^aci^ KO mice, Yoda1, GsMTx4	[16]
	Tight junction	Piezo1 over‐activation damages the expression of TJ protein claudin‐1 through ROCK1/2 pathway and deteriorates the functions of intestinal epithelial barrier and permeability.	Mouse, human	Piezo1 shRNA, Piezo1 sgRNA, ruthenium red, GsMTx4	[17]
Goblet cells	Mucin2 production and secretion	Piezo1 mediates mechanical stimulations and inhibits the methylation of H3K9me3 in goblet cells to promote mucin2 production and secretion.	Mouse, human	Hydrostatic pressures, Piezo1^ΔGC^ mice, Piezo1 siRNA, Yoda1	[18]
	Intestinal motility change	Piezo1 deletion decreases the renewal capacity of the colon stem cells, leading to reduced goblet cells, decreased thickness of mucous layer, and deteriorated intestinal motility.	Mouse, human	Piezo1^ΔGC^ mice	[19]
	Maintain intestinal microbiome	Piezo1 deletion weakens mucus layer ability of confining fecal microbiota and causes alteration of α‐diversity in FAM and MAM.	Mouse, human	Piezo1^ΔGC^ mice, Piezo1 siRNA	[20]
Intestinal stem cells	Proliferation, differentiation	Piezo1 induces cytosolic Ca^2+^ increase and phosphorylates ERK1/2 to promote Drosophila midgut stem cells proliferation and differentiation.	Drosophila	Piezo^KO^ or Piezo^RNAi^ mice	[21]
		Piezo channels participate in inflation‐driven stem cell zone fission in intestinal organoids.	Human	GdCl3	[22]
		The change in the viscoelasticity of the extracellular matrix activates Piezo‐p38 MAPK‐YAP pathway to induce stem cell self‐renewal in intestinal organoids.	Human	Carbon nanotubes	[23]
Enterochromaffin cells	Regulate 5‐HT secretion in response to force	Piezo2 is abundantly expressed in human's and mice's enterochromaffin cells and its activation by force increases extracellular Ca^2+^ and 5‐HT release.	Mouse, human	Piezo2 siRNA, Gd^3+^, GsMTx4	[6]
		Piezo1 functions as a natural ligand to fecal bacteria‐derived ssRNA and mediates systemic 5‐HT secretion	Mouse	Cyclic stretching, Piezo1 siRNA, Piezo1 sgRNA, Yoda1, Villin‐Piezo1^flox/flox^ mice	[24]
Fibroblast reticular cells	Promote lymphocytes recruitment	Piezo1 transfers mechanical signals of fluid flow change and contributes to proper alignment of fibroblast reticular cells in intestinal Peyer's patch and its deficiency results in impaired recruitment of lymphocytes.	Mouse	*Ccl19*‐P1cKO mice	[25]

### 1.1 Impact of Piezo1 on intestinal barrier

As a hollow organ that transports food and other substances, the intestine frequently encounters pathogenic invasions. Consequently, the intestinal barrier (a multilayered structure) serves as a substantial defense to protect against bacteria and other external antigens.^29^ This complex structure, whose components potentially interact with mechanical forces and Piezo channels, comprises the cellular components of epithelia, the mucous layer, the commensal microbiome, and antibacterial products secreted by Paneth cells or enterocytes.^30^, ^31^, ^32^

### 1.2 Mucous barrier

The mucus layer is primarily composed of mucins, high‐molecule‐weight glycoproteins synthesized and secreted by goblet cells, which can bind water to form a gel‐like structure.^33^ Mucins are classified into membrane‐associated and secreted mucins.^34^ Mucins play a critical role in protection, transportation, and hydration in the mucous layer by avoiding straight contact between exotic antigens, especially potentially pathogenic microorganisms.^33^ Mucin2 is regarded as a most crucial member of the secreted mucins in healthy mice and dominant in mucous barrier formation and maintenance, and the deficiency of which is associated with spontaneous inflammation and elevated infection susceptibility.^5^, ^35^, ^36^ Mucin2 synthesis and secretion, including regulated secretion and compound exocytosis, are modulated by microbial stimuli and peristaltic intestinal movements,^37^ both of which are believed to be activated by Ca^2+^.^38^

In goblet cells, several ion channels including the transient receptor potential family, toll‐like receptor family, and acid‐sensing ion channels respond to mechanical stimulations, among which Piezo1 is expressed at significantly higher levels, suggesting its potential role in responding to intestinal mechanical signals and regulating mucin2 secretion during food transportation and digestion.^18^ In an intestinal perfusion model simulating hydrostatic pressure stimulation, changes in mucous layer thickness showed a parallel effect with hydrostatic pressure and the expression of Piezo1 in intestinal tissues.^18^ This research confirmed that Piezo1 responded to a certain intensity of traction and shear stimulation to induce mucin2 secretion in vivo and in vitro via Ca^2+^ influx. Further studies on intestinal goblet cell Piezo1‐deficient mice (Piezo1^△GC^ mice) revealed that Piezo1 activation in goblet cells caused a downstream inhibition of Histone 3 lysine 9‐trimethylation (H3K9me3), a modality of DNA methylation suppressing mucin2 gene transcription, thereby promoting the expression of mucin2 in goblet cells.^19^

Notably, the same team also found that intestinal motility weakened in Piezo1^ΔGC^ mice, characterized by decreased and delayed fecal excretion, along with reduced mucous layer thickness and a decreased number of goblet cells, which were associated with reduced lubrication due to mucous layer impairment. Piezo1 deficiency impaired the self‐renewal capacity of colon stem cells through reducing Wnt and Notch signaling and promoting the enrichment of apoptotic pathway genes, leading to a sharp reduction of goblet cells and mucin secretion, decreased intestinal motility, and even constipation and pseudo‐obstruction.^39^ In summary, Piezo1 in goblet cells responds to different mechanical forces to regulate mucous barrier thickness, stability, and intestinal motility to resist and eliminate foreign antigens. The critical role of Piezo1 in transducing mechanical signals of intestinal peristalsis is confirmed, but the underlying mechanisms still remain to be further explored. The opening of Piezo1 promotes Ca2+ influx, which induces many downstream pathways and may provide many more chances to interfere intestinal functions.

### 1.3 Epithelial barrier

Epithelial cells and protein complexes known as tight junctions (TJs) binding between enterocytes form the epithelial barrier that blocks antigens.^40^ A stable cell count and regular renewal are essential for maintaining epithelial layer function. Piezo1, which detects local cellular stretch signals, maintains epithelial homeostasis by extruding overcrowded live cells and promoting proliferation in low‐density areas, indicating its critical role in epithelial cell proliferation and apoptosis.^41^ Epithelial cells undergo “cell extrusion” to remove dead cells. In this process, cells programmed to die send signals that induce the contraction of an actomyosin ring in surrounding cells, leading to the extrusion of apoptotic cells.^14^, ^42^, ^43^ Similarly, live cells in overcrowded regions undergo extrusion to maintain appropriate density. However, they use a different pathway activated by overcrowding‐induced strain between cells, with Piezo1 participating in this process.^15^ When activated by stretching, Piezo1 maintains homeostatic epithelial cell numbers in low‐density regions by enhancing cell proliferation through ERK1 and promoting a rapid transition from the G2 to the mitosis phase.^44^ Piezo1 is also involved in programmed cellular apoptosis. Under stretch stimulation, Piezo1 activation induces abundant intracellular Ca^2+^ influx, leading to changes in mitochondrial membrane potential and eventual death in nucleus pulposus cells.^16^ Additionally, while Piezo1 maintains cellular Ca^2+^ influx stability by preventing p53‐dependent senescence and apoptosis in steady‐state conditions,^45^ it may also induce abnormal programmed cell death under pathological circumstances. In a study on skeletal stem cells, Piezo1 knockout triggered compensatory upregulation of T‐type voltage‐gated Ca^2+^ channels, activated protein kinase C, and upregulated the expression of Nicotinamide dinucleotide phosphate hydrogen oxidase 4, a reactive oxygen species (ROS)‐generating enzyme, leading to p53‐dependent senescence and apoptosis in skeletal muscle stem cells. Piezo1 likely functions in the gastrointestinal tract, as its role in maintaining epithelial homeostasis has been verified.^15^, ^44^ However, a recent study on pancreatitis suggested that activated Piezo1 mediated Ca^2+^ influx overload, which increases cytotoxicity, causes chronic pancreatic acinar cell death, and ultimately leads to inflammation.^46^ Piezo1 is also associated with ferroptosis, a heated form of programmed cell death that is iron‐dependent and characterized by overaccumulation of lipid peroxides.^47^ In a study on necrotizing enterocolitis, a neonatal intestinal inflammatory disease, ferroptosis was observed in the intestinal tissues of patients with necrotizing enterocolitis, along with the upregulation of acetyl co‐enzyme A synthetase long‐chain family member 4,^48^ a potential Piezo1 downstream protein in in vivo and in vitro experiments,^49^ indicating Piezo1's potential as the next site to interfere necrotizing enterocolitis in early stage and reverse the possible outcomes such as intestinal resection, shock and newborn death of NEC.

Piezo1 was initially identified in *Drosophila* midgut stem cells, a formerly ideal alternative model to study adult stem cell behaviors.^21^, ^50^ Piezo1 promotes cell proliferation by inducing intracellular Ca^2+^ influx, followed by ERK1/2 phosphorylation.^51^ Recently, with the development of intestinal organoids, derived from intestinal stem cells and capable of forming complete intestinal crypt structures in vitro, Piezo1 has been found to be abundantly expressed in intestinal stem cells and actively involved in their proliferation and differentiation activities. In the inflation–collapse model established by Tallapragada et al., the essential role of Piezo1 in inflation‐induced stem cell zone fission and crypt formation is demonstrated.^52^, ^53^ Piezo2, the homolog of Piezo1, is also involved in the proliferation and differentiation of intestinal stem cells. In intestinal organoids, Piezo2 is upregulated by changes in the extracellular matrix (ECM) changes and promotes the downstream p38‐YAP pathway,^22^ which is widely known for regulating cell proliferation, differentiation, and regeneration.^23^

In addition to epithelial cell number and density, the completeness and proper functioning of tight junctions (TJs) are crucial for maintaining the epithelial barrier. Piezo1 in primary human small airway epithelial cells, when activated by extra pressure, massively increases intracellular Ca^2+^ concentration. This activation initiates the calpain cascade, resulting in the degradation of TJ proteins including occludin, Zonula occludens‐1, and Claudin‐18.^54^ Similarly, in the intestines, overexpression of Piezo1 significantly decreases claudin‐1 expression, possibly via the ROCK1/2 signaling pathway, thereby reducing the transepithelial electronic resistance (TEER) and enhancing 4 kDa FITC‐dextran (FD4) permeability, representing a deterioration of the intestinal epithelial barrier and elevation permeability.^17^

**FIGURE 1:**
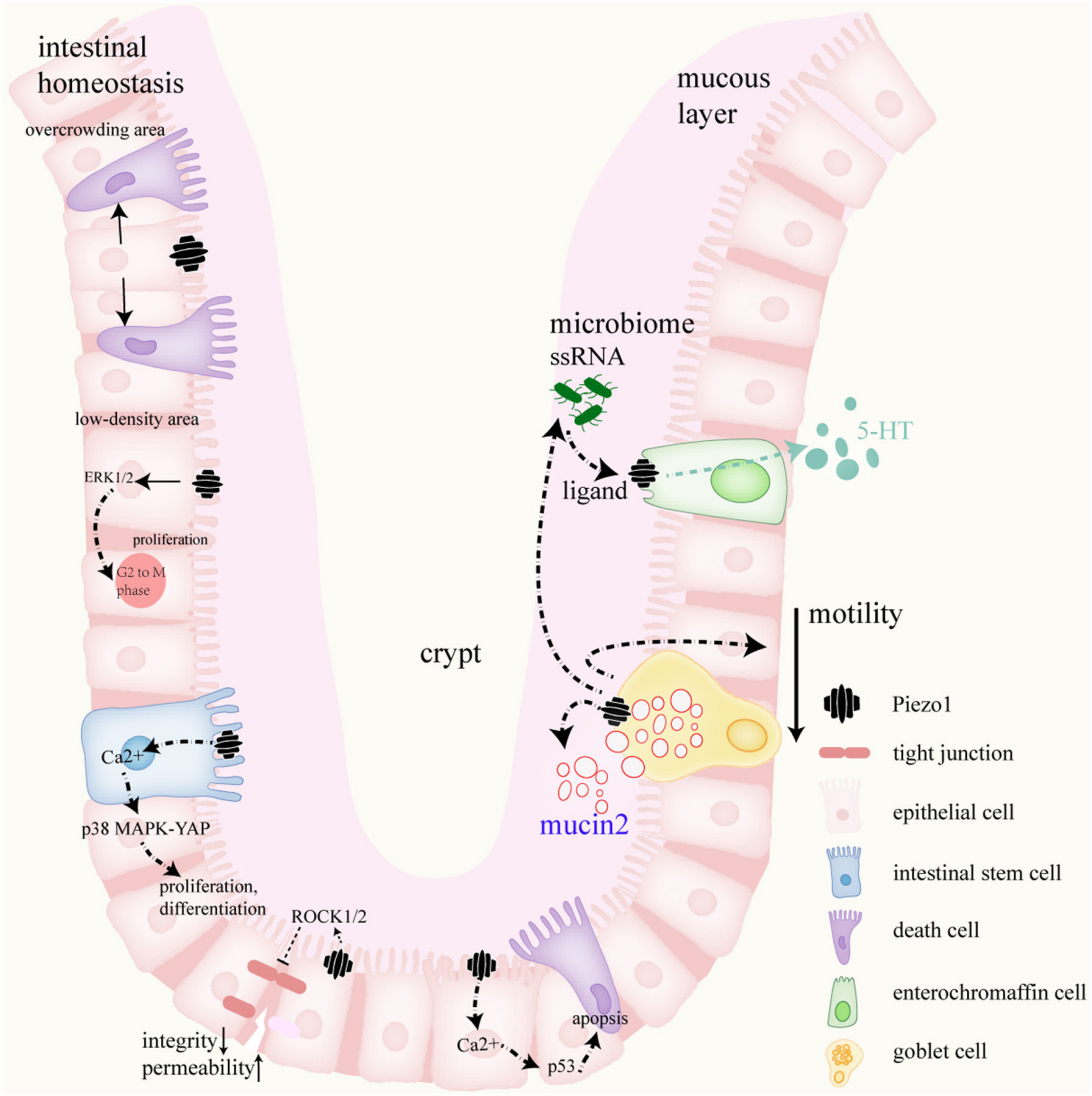
Structure of intestinal epithelia and Piezo1's functions. Piezo1 participates in maintaining the integrity of intestinal barriers in many cell types. In epithelial cells, Piezo1 manages critical cell life activities including proliferation and apoptosis, and also maintains regional cell count homeostasis. Piezo1 activation is associated with intestinal stem cell proliferation and differentiation. In goblet cells, Piezo1 is involved in mucin2 expression and secretion, helps to form the mucous layer, and regulates intestinal motility and microbiome composition. In enterochromaffin cells, Piezo1 acts as a ligand to fecal ssRNA to regulate 5‐HT secretion.

In summary, Piezo1 maintains epithelial cell homeostasis by sensing epithelial cell density and regulating both proliferation and differentiation. Consequently, Piezo1 protects epithelial barrier integrity against the infiltration of harmful pathogens. However, overactivation of Piezo1 significantly reduces the expression of TJs, subsequently damaging the epithelial barrier and its function of permeability. Piezo1 is expressed in many other cell types in the epithelial layer such as Paneth cells, endocrine cells, and intestinal neural ligands, indicating Piezo1 may participate broader and deeper in cell biological activities, making it a potential site to treat intestinal diseases including inflammations, tumors, and motility diseases like irritable bowel syndrome. More future efforts should be made to clarify its various functions in the intestine (Figure 1).

### 1.4 Interaction with commensal microbiome

Due to its widespread expression throughout the digestive tract, Piezo1 can participate in biological activities of specific cell types, such as Paneth cells, enteroendocrine cells, and M cells. Piezo2 is mainly expressed in enterochromaffin cells (EC),^6^ where approximately 95% of serotonin (5‐hydroxytryptamine, 5‐HT) is secreted.^55^ Piezo2 has been reported to transduce mechanical signals to regulate 5‐HT secretion by inducing extracellular Ca^2+^ influx.^6^, ^56^ Sugisawa et al. found that villin‐specific Piezo1 deficiency reduced colitis in mice, as indicated by reduced diarrhea and improved survival, and decreased systemic 5‐HT levels, a key systemic mediator of inflammation.^57^, ^58^, ^59^, ^60^, ^61^ In vivo *and* in vitro experiments further showed that fecal single‐stranded ribonucleic acid (ssRNA) might act as an independent ligand of Piezo1 in enterochromaffin cells, thereby regulating the secretion of 5‐HT.^2^ Nickolls et al. used Neuro 2A and human embryonic kidney293 cells, which naturally express Piezo1 to verify the fecal ssRNA function in Piezo1 activation.^55^ No detectable increase in Ca^2+^ or measurable delay in inactivation was observed in the cell line. However, they found that ssRNA could stimulate Ca^2+^ influx independently of Piezo1, relying primarily on the calcium activity of the cell lines used, thus raising the debate on the interaction between Piezo1 and ssRNA. Nickolls et al. also confirmed that RIN14B, an enterochromaffin model cell line, responded to fecal ssRNA but failed to connect it to Piezo1, suggesting the involvement of other ion channel candidates in intestinal epithelia, such as Piezo2. However, further studies are needed to validate and elucidate these findings.

Piezo1 activity has been recently associated with altered intestinal microbiota in Piezo1^ΔGC^ mice.^24^ Liu et al. found that the α‐diversity was significantly decreased in fecal‐associated microbiota (FAM), a condition often associated with inflammatory bowel diseases,^39^ but increased in mucosa‐associated microbiota (MAM) compared with Piezo^+/+^ mice, indicating a higher risk of potential pathogenic and mobile bacterial infections. Piezo1^ΔGC^ mice exhibited a different bacterial composition, as the ratio of *Firmicutes/Bacteroidota* (F/B), corresponding to inflammatory bowel diseases (IBD) and pathogenic microorganisms such as *Helicobacter hepaticu*s and *Escherichia‐Shigella* increased and short‐chain fatty acid‐producing bacteria such as *Ruminococcus* and *Akkermansia muciniphila* decreased. This indicates that Piezo1 might be crucial in maintaining the intestinal microbiome's normal composition and function and as such may be associated with many inflammatory diseases in the intestine. However, the underlying mechanisms and effects remain to be identified.^20^

## 2 IMPACT OF PIEZO1 ON INTESTINAL IMMUNE RESPONSES

An immune response involving several cellular and molecular events, which can be described as inducers, sensors, mediators, and effectors from different systems, is initiated when the physical barrier of the intestine fails to prevent harmful stimuli from entering.^62^ In general, inflammation is initiated within a controlled range, resulting in increased vascular permeability, leukocyte recruitment, and pro‐inflammatory cytokine release to eliminate harmful triggers and restore tissue homeostasis.^63^ Piezo1 is intricately involved in every stage of the immune response from induction to effectors.

### 2.1 Innate immune system

Intestinal inflammation is often triggered by pathogenic invasion and progresses as pathogens produce immunogenic antigens and secrete pathogenic factors. Pathogen‐associated molecular patterns (PAMPs) from pathogens are recognized by antigen‐presenting cells after crossing the intestinal barrier and binding to specific membrane receptors, such as the family of Toll‐like receptors (TLR) and nucleotide‐binding oligomerization domains. This process further triggers innate immune cells to remove these invaders by releasing cytotoxins or engulfing them, initiating adaptive immune responses such as T‐cell differentiation. Endogenous signals, known as damage‐associated molecular patterns (DAMPs), indicate tissue injury or cell death and can initiate an inflammatory response.^64^, ^65^ Piezo1 is involved in the intestinal inflammatory response induced by both PAMPs and DAMPs. Piezo1 responds to epithelium membrane ruffles induced by bacterial invasion, subsequently affecting adenosine triphosphate (ATP) secretion—a critical mediator of DAMP—and pro‐inflammatory gene transcription.^66^ Upon bacterial infection, Piezo1 forms a complex with TLR4 in macrophages and mediates the calmodulin‐dependent kinase II macrophage stimulating Mst1 and 2 (CaMKII‐Mst1/2) pathway, regulating cytoskeleton remodeling and ROS production.^67^ Piezo1 deficiency accelerates epithelial wound closure, weakens inflammatory responses, and reduces post‐wounding survival by decreasing epithelial Ca^2+^ levels and ROS production.^68^

Piezo1 activates and polarizes macrophages, thereby enhancing their pathogen‐killing ability. Macrophage activity can change in response to certain stimuli, including external mediators and mechanical factors, such as tissue stiffness and matrix architecture,^69^, ^70^ and polarize into pro‐inflammatory (M1) or anti‐inflammatory (M2) phenotypes. Piezo1, a mechanosensitive ion channel, is highly expressed in bone marrow‐derived macrophages,^67^ much more abundantly than any other mechanosensitive ion channel, suggesting a potential relationship with macrophage function. In a dextran sulfate sodium (DSS)‐induced colitis murine model, Piezo1 knockout in macrophages reduced diarrhea and increased survival rates, while the Piezo1 agonist Yoda1 exacerbated colon inflammation.^71^ Piezo1 regulates the macrophage phagocytic function by forming the Piezo1‐TLR4 complex and activating the downstream CaMKII‐Mst1/2‐Rac pathway under lipopolysaccharide (LPS) stimulation; bacterial phagocytosis and clearance are impaired in Piezo1 deficiency bone marrow‐derived macrophages while Yoda1 activated Piezo1 enhanced the phagocytic activity.^67^ Further studies showed that the Piezo1 deficiency results in deficient filopodia and actin formation in response to bacterial invasion and cellular stiffness, highlighting the importance of Piezo1 in macrophage activation during the immune response. Notably, the relationship between Piezo1 and microenvironment “stiffness” seemed to be bidirectional, as the change in cellular or ECM stiffness caused by inflammation activated Piezo1, which is required for cytoskeletal and cellular stiffness formation. Atcha et al.'s study also observed that Piezo1 senses the stiffness of the extracellular stiffness and perceives intracellular actin density, which changes with the increasing stiffness of cultured surfaces. This effect was inhibited in Piezo1^ΔLyzM^ macrophages.^27^

**FIGURE 2:**
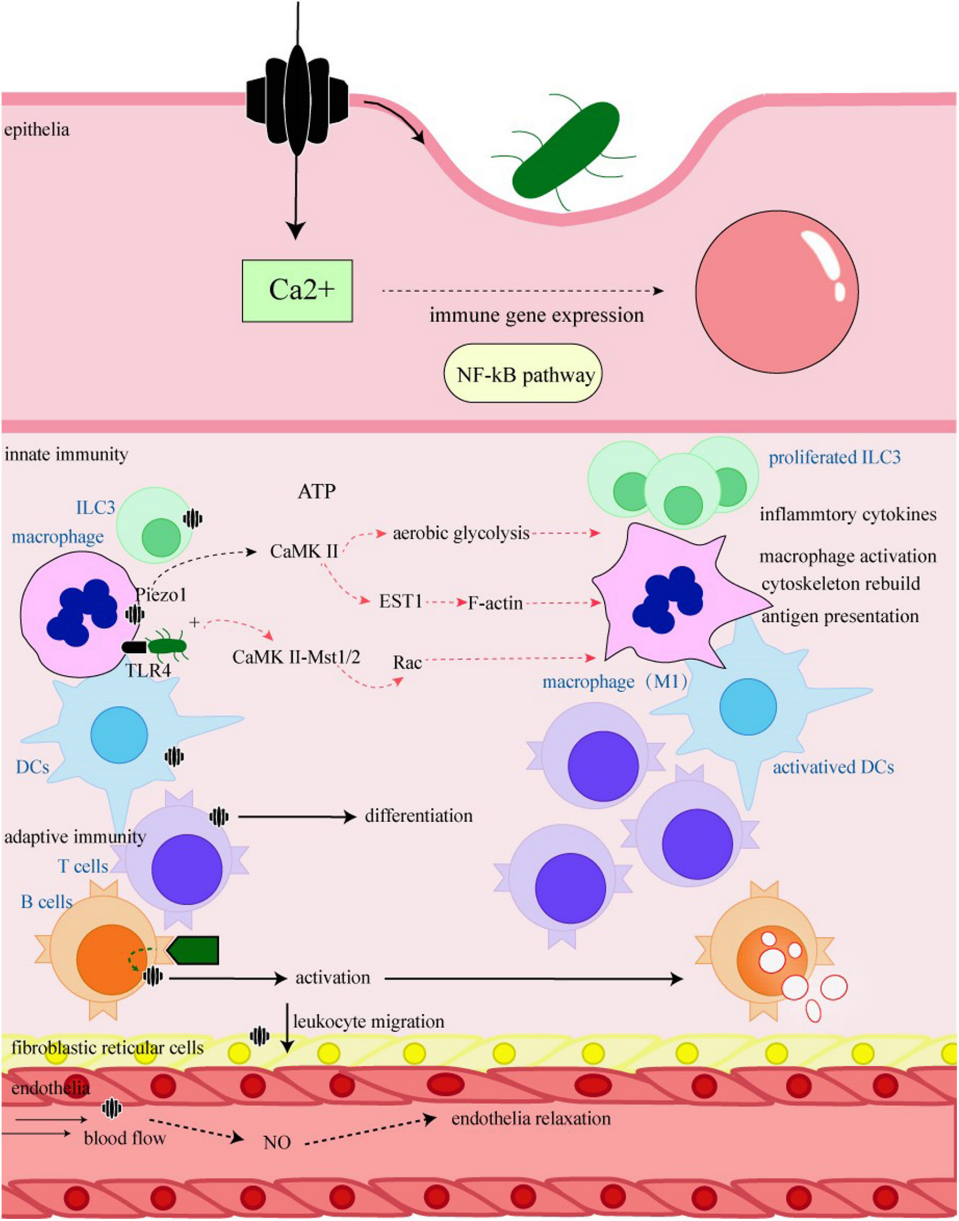
Piezo1's effects in the inflammatory process in different systems. Once pathogens invade epithelia, Piezo1 senses the membrane ruffle and induces Ca2+ influx to activate immune gene expression, the NF‐kB signaling pathway, and the secretion of ATP to further induce inflammation. Once the foreign antigens pass through epithelial cells, Piezo1 participates in the initiation of the immune response. In the innate immunity system, Piezo1 is associated with antigen presentation and the activation of innate immune cells, such as ILC3, dendritic cells, and macrophages by cytoskeleton remodeling, cell activation, and inflammatory cytokine release. In the adaptive immunity response, Piezo1 participates in the activation of both T cells and B cells and helps T‐cell differentiation. Furthermore, Piezo1 induces leukocyte migration, angiogenesis due to hemodynamic change, and lymph changes to confirm and even spread effective inflammation.

Macrophage activation induces inflammation, and Piezo1 plays a key role in this process. Macrophages with Piezo1 deficiency exhibit reduced aerobic glycolysis and impaired downstream inflammatory cytokine secretion under LPS stimulation, while Yoda1 pretreatment upregulates these processes, potentially regulated by Ca^2+^ influx through the calmodulin‐dependent kinase II hypoxia‐inducible factor 1 (CaMKII‐HIF)‐α signals.^71^ Piezo1 in macrophages induced by shear stress is also linked to the activation of the nucleotide‐binding domain, leucine‐rich‐containing family, and pyrin domain‐containing‐3 (NLRP3) inflammasome, which regulates the inflammatory response through IKKβ upregulation.^72^ Furthermore, Piezo1 activation induces the nuclear factor kappa B(NF‐κB) pathway and the abundant release of downstream pro‐inflammatory cytokines (including tumor necrotic factor‐alpha, interleukin [IL]‐6), mediated by Ca^2+^ influx (Figure 2).

Liu et al. found that IL‐6 production by dendritic cells was significantly decreased by GsMTx4 (a specific inhibitor of Piezo1) interference. In contrast, no relationship was found between Piezo1 and T or regulatory T (T‐reg) cells, suggesting that Piezo1 primarily affected innate immune cells in experimental colitis.^73^ The authors revealed that Piezo1 was associated with group 3 innate lymphoid cells (ILC3s) activation, the dominant population in intestinal lymphoid cells.^74^ They identified Piezo1 expression in ILC3s and found that it positively regulates the production of pro‐inflammatory cytokine IL‐17A by promoting the activation and proliferation of ILC3.^75^ Piezo1^−/−^ myeloid cells also failed to sense and transduce pressure‐mediated signals, indicating that Piezo1 is essential for signal transduction in leukocytes originating from myeloid cells.^75^ In addition, dendritic cells exhibited excessively high proliferation, activation, cytokine production, and a significant increase in glucose metabolism when exposed to high rigidity in vitro, with Piezo1 activated by Yoda1, suggesting Piezo1's role in mechanosensitive signal transduction in dendritic cells.^76^ Piezo1 may have similar effects in other innate immune cells, but further studies are required.

### 2.2 Adaptive immune system

Piezo1 participates in the adaptive immune response, including the activation and differentiation of T and B cells.^77^, ^78^ Tokusumi et al. first found that Piezo1 positively influenced cellular immune response initiation in *Drosophila* ^79^ and later identified Piezo1 as a promoter of T‐cell receptors (TCRs) activation through Ca^2+^ influx in human T cells.^80^ Piezo1 is involved in a dendritic cell‐mediated activation of cluster of differentiation (CD) 8+ T cells^76^ and in the development and differentiation of CD4+ T cells in tumors.^81^ Additionally, Piezo1 shows a selective tendency to restrain T‐reg cells, thus attenuating experimental autoimmune encephalomyelitis in Piezo1^−/−^ mice.^80^ Moreover, in B cells, Piezo1 senses the contact between B cell receptors and antigens upon recognizing membrane‐presented antigens, thereby inducing B cell activation.^82^ In summary, Piezo1 is deeply involved in the activation and differentiation of T and B cells, promoting the adaptive immunity response to inflammation. Further understanding of its mechanisms in regulating immune responses may lead to improved treatment for gastrointestinal inflammatory diseases and other conditions.

## 3 IMPACT OF PIEZO1 ON THE VASCULATURE AND LEUKOCYTE MIGRATION

Circulation is crucial in inflammation progression. The abundant blood vessels in the gut transport immune cells, and cytokines, which block and eliminate pathogens, thereby accelerating and spreading inflammation. However, abnormal or immature angiogenesis, which disrupts stability and permeability, exacerbates inflammation by compromising the nutrient supply and impairing the immune response. This process perpetuates inflammation due to the vulnerability of the endothelium.

### 3.1 Angiogenesis and circulation remodeling

Pathological angiogenesis and vascular remodeling are key events in the progression of intestinal inflammation and are commonly observed in patients with inflammatory bowel disease (IBD). The immaturity, fragility, and hypersensitivity of these new vessels facilitate the recruitment and infiltration of inflammatory cells, thus promoting inflammation.^83^ Piezo1, a mechanosensitive ion channel, is associated with endothelial cell angiogenesis and functions. In patients with dextran sulfate sodium (DSS)‐induced colitis, Piezo1 is activated by abnormal blood flow.^84^ Conversely, Piezo1 knockout suppresses lumen formation and reduces the number and quality of newly formed endothelial cells and sprouts.^85^ Activated Piezo1 enhances mitochondrial respiration and glycolysis.^86^ However, in patients with DSS‐induced colitis, pathological angiogenesis is significantly reduced due to attenuated intestinal glycolysis.^87^, ^88^ These studies suggest that Piezo1 activated by shear stress promotes angiogenesis in gastrointestinal inflammation by reducing glycolysis. Conversely, another study using hindlimb ischemia mouse models revealed that Piezo1 in macrophages, when activated by stiffness, deteriorated angiogenesis, and perfusion recovery both in vivo and in vitro in a hypoxic environment,^89^ thereby worsening inflammation. This suggests that Piezo1's effect on angiogenesis may be bidirectional, and its triggers might be more complex than previously understood.

### 3.2 Piezo1 as a sensor of hemodynamic and lymph circulation changes

The mechanosensitive properties and high expression of Piezo1 in the endothelium indicate that Piezo1 is a promising candidate for mechanosensory involvement in the cardiovascular system baroreflex control, participating in the rapid, real‐time feedback regulation of blood pressure.^90^ A study of liver portal veins demonstrated that activated Piezo1 independently induced nitric oxide synthase and endothelium‐dependent relaxation, highlighting its vital role in regulating vascular tension and endothelial cell function.^91^ Beyond blood circulation, Piezo1 similarly affects lymphatic circulation. Chang et al. discovered that in intestinal Peyer's patches, which contain a specialized lymphatic flow system, Piezo1 responds to internal fluid flow in fibroblastic reticular cells.^25^ Piezo1 deficiency causes structural changes in fibroblastic reticular cells surrounding vessels, consequently impairing lymphocyte entry and mucosal immune response.

### 3.3 Piezo1 may be associated with distant migration of cells

The distant migration and infiltration of immune cells into the intestine are crucial components of inflammatory progression, and there is a possible connection to Piezo1. When activated by shear force, Piezo1 further activates Calpain in endothelial cells, leading to focal adhesion disassembly and angiogenesis.^92^ Piezo1 channel activation and Ca^2+^ influx promote endothelial cell migration during embryonic angiogenesis,^93^ suggesting Piezo1's underlying role in cell migration and potential involvement in metastatic cancer. Previous studies have shown that Piezo1 is tightly associated with gastric cancer cell metastasis and proliferation,^94^, ^95^ with relatively higher expression in gastric cancer tissues with omentum metastasis and metastatic lymph node tissues.^96^ During cancer metastasis, the process where epithelial cells lose polarization and adhesion while acquiring infiltration and migration characteristics of mesenchymal cells called epithelial‐mesenchymal transition (EMT) is crucial in inducing metastasis. Wang et al. found that Piezo1, when activated by Yoda1 in gastric cancer cells, induced high expression of EMT‐related proteins, including Vimentin, HIF‐1α, vascular endothelial growth factor, and N‐cadherin. Piezo1 interacted with HIF‐1α and upregulated Calpain1/2 to accelerate the EMT and cell migration, thereby promoting distant metastasis.^96^ Similarly, Piezo1 may affect intestinal epithelial cells and alter epithelium infiltration or accelerate cell migration, which warrants further studies in this specific field.

## 4 CONCLUSION

The ion channel Piezo1, widely distributed and highly sensitive to mechanical forces, is involved in many intestinal inflammatory events. Piezo1 efficiently regulates the physical intestinal barrier, protecting intestinal tissues from external threats. Piezo1 extrudes live cells in overcrowded areas and promotes intestinal stem cell proliferation and differentiation in low‐density regions, maintaining epithelial homeostasis. Its effect on goblet cells ensures that the mucous layer and intestinal motility function normally. Additionally, Piezo1 responds to foreign antigen invasion by initiating an inflammatory response and activating immune cells such as dendritic cells and macrophages. Moreover, Piezo1 regulates hemodynamic changes, angiogenesis, and cytokine secretion while promoting abnormal cell apoptosis and inflammation.

Although the importance of Piezo1 in intestinal inflammation has been acknowledged, the exact mechanisms underlying its regulation remain unclear. Therefore, further exploration of the clinical translation of Piezo1 in specific intestinal microenvironments is necessary to identify potential targets for intestinal inflammation treatment.

## AUTHOR CONTRIBUTIONS

Qinlei Jiang, Zhenyu Li, Dan Dang, and Hui Wu contributed to conception, drafting, and revision of the manuscript. Qinlei Jiang, Zhenyu Li, and Jiaqi Wei were responsible for bibliographic retrieval. All authors have read and agreed to the published version of the manuscript.

## FUNDING INFORMATION

National Natural Science Foundation of China (82271737, 82301952); Jilin Provincial Department of Science and Technology (20230204080YY).

## DISCLOSURES

The authors declare that they have no competing interests.

## ETHICS STATEMENT

Not applicable.

## CONSENT FOR PUBLICATION

Not applicable.

## Data Availability

Not applicable.
